# Evaluation of GKN1 and GKN2 gene expression as a biomarker of gastric cancer 

**Published:** 2018

**Authors:** Fatemeh Dokhaee, Sogol Mazhari, Mohammad Galehdari, Ayad Bahadori Monfared, Kaveh Baghaei

**Affiliations:** 1 *Faculty of Biological Sciences, North Tehran Branch, Islamic Azad University, Tehran, Iran*; 2 *Basic and Molecular Epidemiology of Gastrointestinal Disorders Research Center, Research Institute for Gastroenterology and Liver Diseases, Shahid Beheshti University of Medical Science, Tehran, Iran*; 3 *Faculty of Medicine, Shahid Beheshti University of Medical Sciences, Tehran, Iran*; 4 *Gastroenterology and Liver Diseases Research Center, Research Institute for Gastroenterology and Liver Diseases, Shahid Beheshti University of Medical Sciences, Tehran, Iran *

**Keywords:** Gastric cancer, Gastrokine-1(GKN1), Gastrokine-2 (GKN2), Real-time PCR

## Abstract

**Aim:**

The aim of this study was to investigate the expression of GKN1 and GKN2 genes as probable biomarkers for gastric cancer.

**Background:**

Gastric cancer is a multifactorial process characterized by the uncontrolled growth and dissemination of abnormal cells. Survival rates of gastric cancer tend to be poor, a plausible explanation is a combination of a late-stage diagnosis and limited access to treatment. In this regard, finding relevant and measurable biomarkers is urgently needed.

**Methods:**

27 samples of gastric cancer tissues were enrolled into this study, according to their pathological responses. The alteration of genes expression were evaluated by Real-Time PCR technique.

**Results:**

Our findings showed the significant reduction of Gastrokin-1 and Gastrokine-2 genes expression in the cancerous specimens in comparison with the normal tissues. (P = 0.008 and P = 0.004 respectively).

**Conclusion:**

Our findings showed the significant reduction of Gastrokin-1 and Gastrokine-2 genes expression in the cancerous specimens in comparison with the normal tissues. (P = 0.008 and P = 0.004 respectively).

## Introduction

 Gastric cancer (GC) is the second leading cause of death associated with cancer (1). In contrast with the reduction of the incidence of GC in the most developed nations, in some developing countries the high prevalence is observed. GC is a multi-factorial disease that caused by the presence of infectious agents, genetic and environmental subjects. Hence, extraterritorial studies are not probable to be cited in the other population (2). According to investigations, the incidence of GC in Iran is 1.49 in men and 25.9 in women per 100,000, so Iran is considered a high-risk country (3-5). 

In the traditional category (Lauren), GC is divided into two categories: intestinal and diffuse types. Environmental factors and infection with Helicobacter* pylori (H.pylori)* may cause intestinal type, which is more benign, whereas genetic abnormalities are related more to diffuse type of disease, as the resistant type (6).

 Due to the poor symptoms at the early stages of GC, most patients’ carcinogenesis is diagnosed with metastasis in the advanced stages, so the overall clinical outcome of GC patients remains unsatisfactory (7). In this regard, substantial studies have been done on the expression of genes associated with GC in order to improve early detection of this fatal disease. Gastrokine 1 (GKN1), and Gastrokine 2 (GKN2), are belonging to Gastrokine family, which are expressed noticeably in the normal tissue of the gastric epithelium and play a significant role in maintaining the integrity and homeostasis of gastric mucosa (8). 

*GKN1*, also called antral mucosal protein (AMP)-18, CA11, FOVEOLIN, is a protein by molecular weight of 18 kDa, with 'cytokine-like' activity which is synthesized by the cells of the antral gastric mucosa (9). The level of *GKN1* expression in the other sections of the gastrointestinal system and GC cell lines have been completely stopped. It is noteworthy that its expression in the inflammation zones caused by *H.pylori *is reduced as well (10). *GKN1* reduces cell viability, proliferation, and colony formation by inhibiting cell cycle development and epigenetic modification by down-regulating the expression levels of DNMT1 and EZH2, and DNMT1 activity, and inducing apoptosis through the death receptor-dependent pathway. Thereby, *GKN1* is involved in inhibition of development and progression of GC (11).

*GKN* family consists three members, *GKN1*, *GKN2*, and *GKN3*. Between them, *GKN2*, also known as, TFIZ1, GDD, and blottin, are mainly studied. *GKN2* is a secretory protein of gastric epithelial cells and its expression is remarkably down regulated or absent in GC tumor tissues (12). In addition, over-expression of *GKN2* significantly inhibits the JAK2/STAT3 signal pathway to further up-regulate Bax, and down-regulating Bcl-2, Cyclin D1, MMP2, and MMP9, therewith resulting in reduced proliferation and invasiveness, enhancement of apoptosis (13).

In the current study, we investigate *GKN1 *and *GKN2* gene expression in Iranian population for the first time, in order to find their association with GC and achieve probable biomarkers. 

## Methods

**Sampling**

This study was performed on twenty-seven tissue samples from patients admitted to the Taleghani Hospital, Tehran. The samples were obtained from GC patients who had undergone surgery. None of the patients received preoperative treatment with chemotherapy, radiotherapy or immunotherapy. A part of each tissue sample was placed in 10% phosphate-buffered neutral formalin for pathologic diagnosis. The other part was immediately stored in RNA Later stabilizing solution (Qiagen, Germany ) at -70 °C for subsequent analysis.

**RNA extraction and cDNA synthesis**

Total RNA was isolated from normal and GC tissues using RNA Purification Mini Kit (FavorGen, Taiwan), according to the manufacturer's instructions. The concentration of RNA extract was measured with the Nanodrop (Thermo Fisher Scientific, USA) by absorbance at 260 nm. Total RNA was converted to cDNA, according to Yekta Tajhiz cDNA synthesis kit by using M-MLV RT enzyme and random hexamer primer. Quality and concentration of cDNA were determined by Nanodrop Device. 

**Quantitative real-time PCR**

Initially, primers for the desired genes (GKN1 and GKN2) and internal control gene (GAPDH) were designed by Primer 3 software. To ensure the accuracy and specificity of the primers, Gene Runner (ver. 6.0.04) was used. Finally, primers were blasted at NCBI. The sequence of the primers is shown in Table 1. To carry out quantitative real-time PCR reaction, using qPCR Master Mix 2X (Yekta tajhiz azma, Taiwan) pursuant to the manufacturer's instructions. (The amount of 10µl SYBER Master Mix, 0.8 µl Forward primer, 0.8 µl Reverse primer,150 ng/µl cDNA and dH_2_O in total volume 20 µl were entered to Real-time PCR reaction.) 

The alteration in mRNA expression’s level were compared with control group. Data were analyzed using using ABI 7500 and REST 2009 software version 2.0.13 and Prism software statistical package version 5 (GraphPad Software, USA). The t-test analsysis was used to determine alteration expression in GC samples compared with normal group. Significance was set as the difference P value is less than 0.01.

**Table 1:** Primer sequence of target and reference genes

Primer	Primer sequence	Gene bank ID
GKN1 Forward	5’CCAAGGGCCTGATGTACTC 3’	NM_019617.3
GKN1 Reverse	5’CTCTTGCATCTCCTCAGCC 3’	
GKN2 Forward	5’GGATCATGCTCTTCTACCAC3’	NM_182536.2
GKN2 Reverse	5’GATTGTTCAGAGGAGGGATGTTC3’	
GAPDH Forward	5’GAAGGTGAAGGTCGGAGTCA3’	NM_001256799.2
GAPDH Reverse	5’AATGAAGGGGTCATTGATCA3’	

## Results

In this study, 27 cancer specimens’ samples were selected to evaluate the altered expression of *GKN1* and *GKN2* genes. Pathological and clinical observation including age of patients, site and type of invasion was determined. The sample consisted of 22 male and 5 female patients between the ages of 28 and 81. The features of the studied sample are shown in table 2.

**Figure 1:**
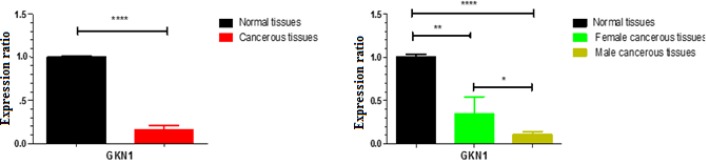
A: Alteration in the *GKN1* gene expression in comparison with normal tissues. The expression was significantly down_regulated by mean factor(-0.8413 ± 0.04999) in the majority of 27 GC cases. GKN1 expression in sample group is different to control group. (P-value<0.0001). B: According to sexuality, *GKN1 *expression shows higher reduction in men

**Figure 2:**
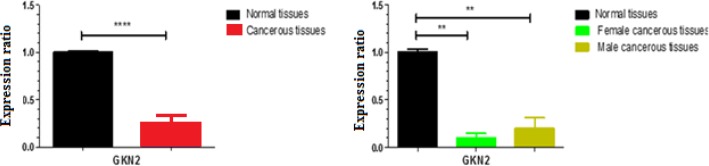
A: The graph is associated with changes in *GKN2* gene expression related to the normal tissues. Overall *GKN2* expression was noticeably decreased by difference in mean factor (-0.8361 ± 0.08537). P-value is reported less than 0.0001.B: There is no significant difference in expression level of *GKN2* in both genders

**Figure 3:**
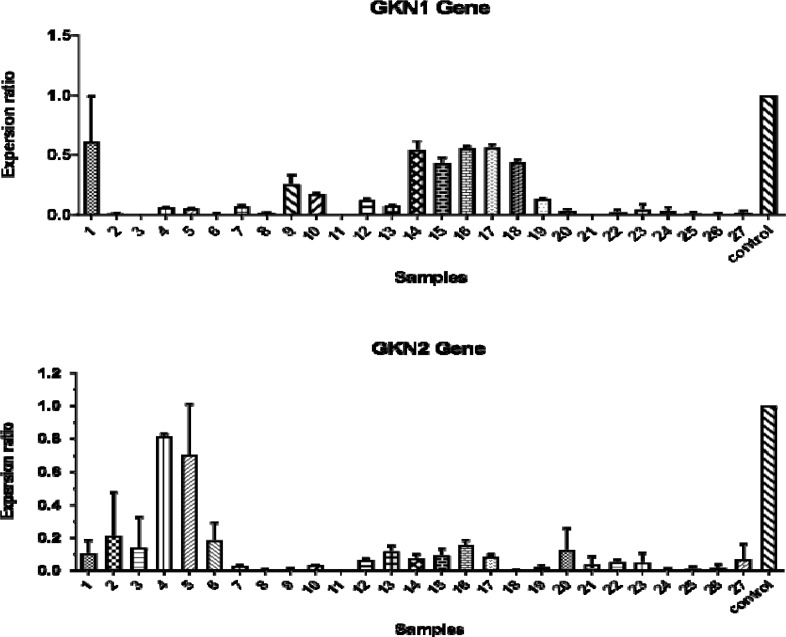
Expression level of GKN1 and GKN2 in patients compared to normal individually

The decrease in expression of* GKN1* and *GKN2* genes in cancerous tissues were observed in all samples in comparison with normal tissue. The absence of *GKN1* and *GKN2 *expression was confirmed at the mRNA level by real time PCR technique on 27 representative cancerous tissues (Figure 1-3).

**Table 2:** Features of the studied samples

Feature		Frequency (%)
Age	> 50 ≤ 50	20 (74%) 7 (36%)
Sex	Women Men	5 (18%) 22 (81%)
Cancer site	Gastric cardia mass Gastric mucosa Gastric body Distal stomach	7 (25.9%) 11 (40.8%) 6 (22.2%) 3 (11.1%)
Cancer type	Intestinal type Diffuse type	18 (67%) 9 (33%)

## Discussion

GC is one of the leading types of cancer that has a high mortality rate. One of the main reasons for this high rate is the inability to diagnose the disease in the early stages. In fact, GC is the second leading cause of death associated with cancer (14). Althogh the statistical difference in prevalance of GC data among geographical distributions is critical, the investigation of national studies is more reliable. Recent studies have shown a significant reduction in the 5-year survival rate of GC patients in Iran (15). Therefore, a deeper understanding of the pathogenesis and biological features of GC is essential for enhancing early detection, treatment methods and patient's longevity. On the other hand, genetic and epigenetic changes play an important role in the GC process. Their effects are caused by altering protein expression levels or change their performance (16). The discovery of new biomarkers and their application, in conjunction with a traditional cancer diagnosis, staging, and prognosis, will help to improve early detection and patient’s care.

*GKN1 *and *GKN2* genes are expressed extensively in the natural tissue of the gastric epithelium. Recent evidence illustrated that *GKN1* and *GKN2* may play an important role in the preservation of gastric mucosal homeostasis and function as a gastric individual tumor suppressor (8). The *GKN1* gene mapped on chromosome 2 at 2p13.3 site, which resulted a protein with a molecular weight of 18 kDa. This protein plays an important role in stability and restoration process of mucus layer (17,18).

In 2003, Toback *et al. *found that* GKN1* is responsible for repair of the gastric’s epithelialium after injuries and is also capable of inhibiting the proliferation of cancerous cells (9). In correlation with previous study, in 2011, JJ Yoon *et al.* examined the inactivation of the *GKN1 *gene in the progression of GC. They pointed out that inactivation of this gene plays an important role in the development of GC as a primary occurrence. They also reported a significant reduction level of the *GKN1* expression in GC cells (19). In 2012, Mao* et al.*, found that non-steroidal drugs and infection can lead to gastric disorders by reducing the expression of the *GKN1 *(20).

On the other hand, in contrast with *GKN2*, there are few investigations about the role of *GKN2* in GC. The *GKN2* gene, is located on chromosome 2 at 2p13.3, that encodes a 18.3-kDa protein (17). Based on the research conducted by Dai *et al. GKN2* reduction or loss of expression was observed in GC cell lines BGC-823, SGC-7901 and AGS (8). Baus-Loncar and colleagues (2007) showed that *GKN2* plays an important role in maintaining gastric mucosal homeostasis by regulating *GKN1* activity (21). The relationship between expression of *GKN1 *and *GKN2* was demonstrated by Am. A.*et al. *in 2015. They reported that there is an adjacent correlation between the expressions of these two genes. Moreover, they stated that there is a significant statistical connection between reduced levels of both *GKN1* and *GKN2* gene expression in GC tissue in comparison with normal tissues (22). Hence, *GKN2* interacts with the *GKN1 *protein and regulates the amount of *GKN1* that results in the formation of sustainability and homeostasis in the epithelial mucus layer.

In the current study, we examined *GKN1 *and *GKN2* gene expression levels in an Iranian population with q-RT PCR method, for the first time. As our expectation, our data, in consistant with the other studies including KA *et al. *and also Rippa *et al. *both showed that the expression of GKN1 gene in the cancerous tissue was reduced (23,24). Another study had been done by Menheniott T.R *et al.* demonstreted the reuction expression in both gastrokines genes in gastric cancer tissues (25). Due to the important roles of *GKN1 *and *GKN2* genes and their relationship, in the gastric mucosal defense mechanism and their gastric tumor suppressor activity,* GKN1* and *GKN2 *might be reliable biomarkers for detecting GC in early stages. In this regard, comprehensive studies including investigation of the relationship between patient indices and changes in expression level of GKN1 and GKN2 are needed.

## Conflict of interests

The authors declare that they have no conflict of interest.
